# Clinical physiology aspects of chloremia in fluid therapy: a systematic review

**DOI:** 10.1186/s13741-020-00171-3

**Published:** 2020-12-10

**Authors:** David Astapenko, Pavel Navratil, Jiri Pouska, Vladimir Cerny

**Affiliations:** 1 Department of Anesthesiology, Resuscitation and Intensive Care Medicine, University Hospital Hradec Kralove, Sokolská 581, 500 05 Hradec Kralove, Czech Republic; 2 Faculty of Medicine in Hradec Kralove, Charles University, Prague, Czech Republic; 3 Department of Urology, University Hospital Hradec Kralove, Hradec Kralove, Czech Republic; 4 Department of Anesthesiology, Resuscitation and Intensive Care Medicine, University Hospital Plzen, Plzen, Czech Republic; 5 Faculty of Medicine in Plzen, Charles University, Plzen, Czech Republic; 6 Department of Anesthesiology, Perioperative and Intensive Care Medicine, Faculty of Healthcare Studies, J. E. Purkyne University in Usti nad Labem and Krajska zdravotni a.s. (Regional Healthcare JSC), Masaryk Hospital in Usti nad Labem, Usti nad Labem, Czech Republic; 7 Center of Research and Development, University Hospital Hradec Kralove, Hradec Kralove, Czech Republic; 8 Department of Anesthesia, Pain Management and Perioperative Medicine, Dalhousie University, Halifax, NS Canada; 9 Technical University in Liberec, Liberec, Czech Republic

**Keywords:** Fluid therapy, Chloride, Hyperchloremia, Metabolic acidosis, Renal failure

## Abstract

**Background:**

This systematic review discusses a clinical physiology aspect of chloride in fluid therapy. Crystalloid solutions are one of the most widely used remedies. While generally used in medicine for almost 190 years, studies focused largely on their safety have only been published since the new millennium. The most widely used solution, normal saline, is most often referred to in this context. Its excessive administration results in hyperchloremic metabolic acidosis with other consequences, including higher mortality rates.

**Methods:**

Original papers and review articles eligible for developing the present paper were identified by searching online in the electronic MEDLINE database. The keywords searched for included hyperchloremia, hypochloremia, and compound words containing the word “chloride,” infusion therapy, metabolic acidosis, renal failure, and review.

**Results:**

A total of 21,758 papers published before 31 May 2020 were identified; of this number, 630 duplicates were removed from the list. Upon excluding articles based on their title or abstract, 1850 papers were screened, of which 63 full-text articles were assessed.

**Conclusions:**

According to the latest medical concepts, dyschloremia (both hyperchloremia and hypochloremia) represents a factor indisputably having a negative effect on selected variables of clinical outcome. As infusion therapy can significantly impact chloride homeostasis of the body, the choice of infusion solutions should always take into account the potentially adverse impact of chloride content on chloremia and organ function.

## Background

This systematic review aims to summarize the state-of-the-art knowledge about the role of chloride and chloremia in the context of infusion therapy with special emphasis on the population of patients receiving intensive and perioperative care.

Infusion therapy involving the administration of ion- or glucose-containing solutions (crystalloid solutions) is one of the most widely used procedures in clinical medicine; in the field of anesthesiology and intensive care medicine, crystalloid solution administration is almost a universal strategy in all patients. While having been employed in medicine for almost 190 years, no studies focused largely on their safety were published before 1999.

In international and intercontinental comparisons, the choice of crystalloid solutions in clinical practice is highly variable. Apart from their hemodynamic and volumetric efficiency, the debate in recent years has been dominated by the issue of their safety, in particular, concerning chloride ion content and their potential impact on biological processes within the body at various levels (cellular, organ, and systemic). In the human body, chloride (Cl^−^) is the most abundant anion playing a crucial role in physiological processes, regulation, and signaling pathways (Berend et al. 2012; Yunos et al. 2012; Bandak and Kashani 2017).

## Methods

Original papers and review articles eligible for developing the present paper were identified by searching online in the electronic MEDLINE database to compose a systematic review. The keywords searched for included hyperchloremia, hypochloremia, and compound words containing the word “chloride,” infusion therapy, metabolic acidosis, renal failure, and review. No time limit was set, and the preferred language was English.

## Results

**Fig. 1:**
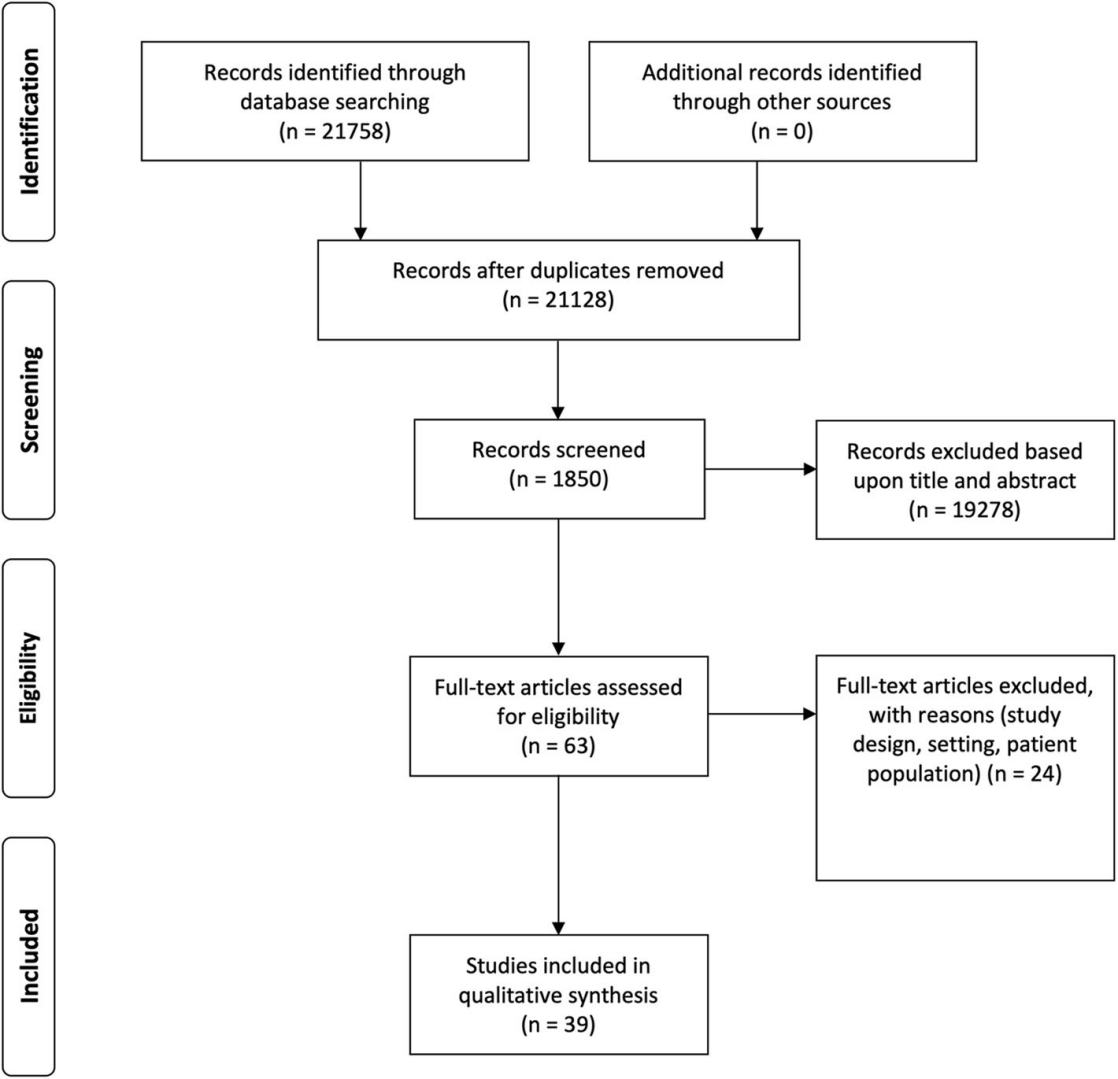
PRISMA flow chart

A total of 21,758 papers published before 31 May 2020 were identified; of this number, 630 duplicates were removed from the list. Upon excluding articles based on their title or abstract, 1850 papers were screened, of which 63 full-text articles were assessed. The relevant publications were subsequently selected by individual authors according to the topic they had been assigned to cover based on their area of expertise. A PRISMA flow chart is shown in Fig. 1. The sorting and selection of articles were done using the online rayyan.qcri.org tool.

## Discussion

### The physiology of chloride in the body

Cl^−^ is the most abundant anion in the serum and the extracellular and intracellular space, making up about 70% of all atoms or groups of atoms carrying negative electronic charges (anions) within the body. The body of an adult contains about 115 g of Cl^-^ which plays important roles in the maintenance of osmotic pressure, acid-base balance, muscle activity, the transport of bodily fluids between individual compartments, maintaining the correct pH value of gastric juice, and it is involved in protein digestion and micronutrient absorption (Berend et al. 2012; Yunos et al. 2010; Koch and Taylor 1992).

Cl^−^ concentrations are actively controlled by several transporters and co-transporters. Its physiological range has been arbitrarily set at 97–107 mmol/L. Cl^−^ transporter and co-transporter mutations are responsible for some diseases such as cystic fibrosis, chronic pancreatitis, epilepsy, and cataract (Puljak and Kilic 2006). The role of all Cl^-^ channels and co-transporters are not fully understood yet (Murek et al. 2010).

**Fig. 2:**
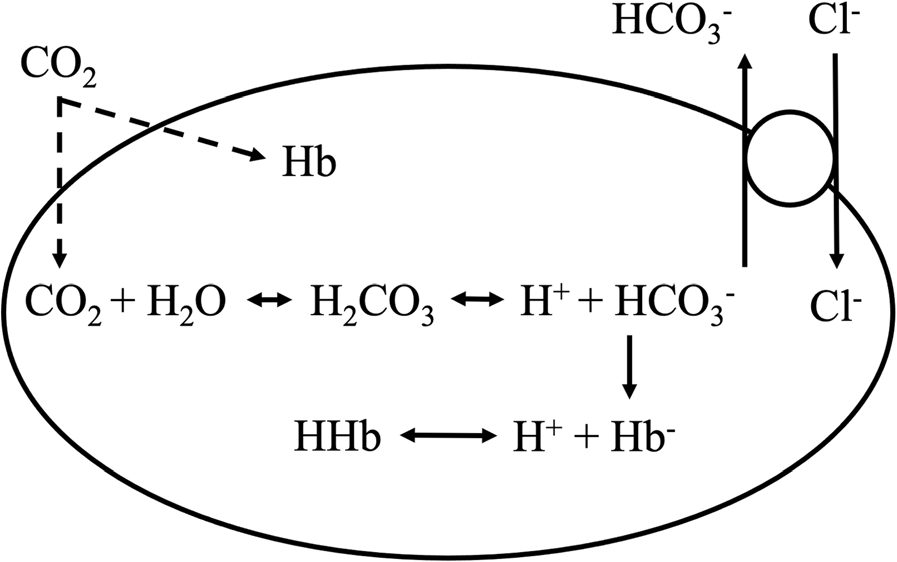
*CO*_2_ carbon dioxide, *H*_2_*CO*_3_ carbonic acid, *HCO*_3_^−^ bicarbonate anion, *H*^+^ hydrogen cation, *Hb*− hemoglobin, *HHb* hydrogen cation bound to hemoglobin, *Cl*^−^ chloride anion

The intracellular Cl^−^ concentrations are dependent on the resting membrane potential. At a resting potential of − 70 mV, average concentrations of Cl^−^ are 2–4 mmol/L. By contrast, the concentrations of Cl^−^ in erythrocytes with a resting membrane potential of − 15 mV are 70 mmol/L. As a result, Cl^−^ can effectively cross the erythrocyte membrane in response to acute changes in the internal milieu on passing from the arterial to the venous end of the capillary. The erythrocyte chloride shift (also known as the Hamburger phenomenon or effect) makes it possible to transport carbon dioxide from tissues in the form of bicarbonate (Fig. 2). As a rule, and, similarly as with hematocrit, erythrocyte chloride concentrations in venous blood are higher than in arterial blood. The erythrocyte chloride shift thus largely contributes to the maintenance of a stable pH during the transport of gas in the blood (Westen and Prange 2003). Abnormal serum Cl^−^ concentrations usually indicate a serious underlying metabolic disorder such as metabolic acidosis or alkalosis, making chloremia, an important part of the assessment of many pathological conditions.

**Fig. 3:**
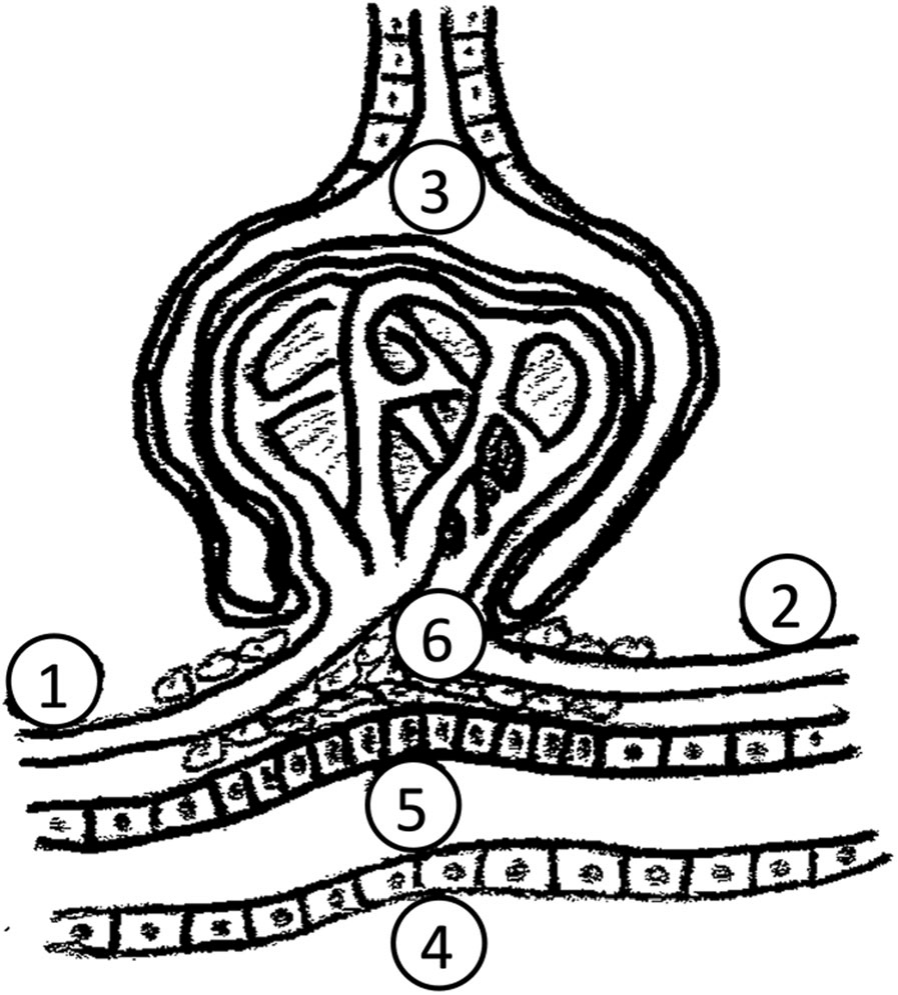
1—*vas afferens*, 2—*vas efferens*, 3—a glomerulus with Bowman’s capsule and primary urine, 4—distal tubule adhering to the glomerulus, 5—*macula densa* cells, 6—mesangial cells

Cl^−^ is excreted primarily by the kidney (approx. 180 mmol/day depending on intake). The bulk of Cl^−^ is actively resorbed in the second proximal tubule segment thus allowing for bicarbonate resorption in the first segment. Chloride is further absorbed in the thick segment of the ascending limb of the loop of Henle by the Na^+^–K^+^–2Cl^-^ co-transporter whereby Cl^−^ concentrations are critical for proper feedback function via the *macula densa* cells of the juxtaglomerular apparatus (Fig. 3). In hypovolemia, Cl^−^ concentrations in the *macula densa* cells decrease thus stimulating endocrine renin secretion and renin-angiotensin-aldosterone axis activation. The result is sodium retention, an increase in blood pressure, and effective filling of the vascular system. Proper fluid management will thus raise Cl^−^ concentrations in the *macula densa* cells while lowering renin secretion (Xu et al. 2011). Blockage of the ion transporter in the loop of Henle represents the pharmacodynamic effect of loop diuretics involving an increase in diuresis (through increased sodium excretion) without an increase in renin secretion (increase in Cl^−^ concentrations in the *macula densa* cells). In the distal tubule, Cl^−^ is resorbed by another co-transporter type (Na^+^–Cl^−^) sensitive to thiazide diuretics. In the distal nephron collecting ducts, Cl^−^ is resorbed upon stimulation by aldosterone. Its increased resorption entails a lack of a negative charge, which naturally opposes the flow of sodium cations resulting in sodium and water retention and the development of hypertension.

Cl^−^ excretion plays an important role in adaptation to metabolic acidosis and chronic respiratory acidosis. The increase in Cl^−^ losses without increased sodium losses makes the *strong ion difference* (SID) greater enabling an increase in bicarbonate concentrations and pH normalization. Stewart’s approach describes the assessment of acid-base balance disorders using SID (see below) (Kimura et al. 2018).

Less is known about the signaling role of Cl^−^. Chloride interacts with many proteins and can directly alter their activity. As mentioned in the case of erythrocytes, the intracellular concentrations of chloride (Cl^−^_[in]_) can be largely variable depending on the type of cells and their stimulation. The electrochemical gradient built across the plasma membrane is maintained primarily by the Na^+^–K^+^ ATP-dependent pump. Upon cell stimulation, the gradient is rapidly altered together with Cl^−^_[in]_. Cl^−^_[in]_ modulates the activity of transcription factors, protein kinases, and other ion transporters (Lüscher et al. 2020).

### Pathophysiology of hyperchloremia in a clinical context

Hyperchloremia is a state of electrolyte imbalance in which the serum chloride anion concentration increases over 107 mmol/L (the value may differ according to the reference limits of the respective laboratory). As such, hyperchloremia has no specific symptoms, which are actually due to the underlying cause. By etiology, hyperchloremia can be divided into 3 types: (i) iatrogenic in fluid therapy with crystalloid solutions, (ii) due to excess loss of water (loss of pure water or higher loss of water compared to chlorides), and (iii) due to increased renal chloride reabsorption (Table 1).

**Table 1:** List of causes of hyperchloremia

Mechanism	Site of action	Example
Chloride intake		Fluid therapy with high-chloride solutions Total parenteral nutrition
Loss of water	Kidney	Diabetes insipidus Diuretics A polyuric phase of renal failure
	Other than the kidney	Fever Hypermetabolism Diarrhea Burns Exercise and severe dehydration
An absolute or relative increase in chloride tubular reabsorption rates		Renal tubular acidosis Renal failure Acetazolamide Reconstructive surgery of lower urinary tract for urine derivation Post-hypocapnia states

Adapted from Bandak G, Kashani KB. Chloride in intensive care units: a key electrolyte. *F1000Res*. 2017; 6:1930

Hyperchloremia frequently develops in critically ill patients following fluid resuscitation with normal saline (NS) (Hammond et al. 2020). An increase in Cl^−^ concentration is accompanied by a decrease in pH giving rise to an acid-base balance disorder, referred to as hyperchloremic metabolic acidosis. The main pathophysiological mechanisms leading to this condition remain a subject of controversy. In the early 1980s, Stewart proposed an innovative model of acid-base balance regulation (Morgan 2009) as he considered changes in Cl^−^ concentrations to be particularly important given the serum electroneutrality and changes in bicarbonate concentrations. According to Stewart, pH is determined by three independent variables: (i) the strong ion difference (SID) as the difference between the sum of all strong cations (Na^+^, K^+^, Ca^2+^, Mg^2+^) and the sum of all strong anions (Cl^−^, lactate, ketones, etc.), (ii) the partial pressure of carbon dioxide (p_a_CO_2_), and (iii) the total concentrations of weak non-volatile acids [A^tot^]—inorganic phosphate and albumin. These variables alter the degree of H_2_O dissociation to H^+^ and OH^−^ according to the basic physicochemical principles such as the law of mass conservation and the electroneutrality principle.

Changes in Cl^−^ concentrations, induced either by regulatory mechanisms or the underlying acid-base balance disorder in the body, can occur independently of the change in Na^+^ concentrations controlled hormonally to maintain plasma osmolality and water balance. Cl^−^ concentrations may alter while crossing cell membranes (consistent with the Gibbs-Donnan membrane equilibrium) and due to changes in renal excretion based on acid-base balance. In hyperchloremia, increased Cl^-^ concentrations decrease SID leading to a decrease in bicarbonate concentrations, an increase in free H^+^ and acidosis.

In the kidney, hyperchloremia causes vasoconstriction of the *vas afferens* and a decrease in the glomerular filtration rate (GFR). In his dog model, Wilcox demonstrated that renal blood flow and GFR are regulated by Cl^−^ concentration. Hyperchloremia causes renal vasoconstriction by inhibiting the intrarenal release of renin and angiotensin II. The decrease in GFR and renal blood flow occurs independently of kidney innervation (Leipziger and Praetorius 2020), increased by previous sodium depletion, and is related to tubular reabsorption mechanisms involving chlorides. This Cl^−^-induced vasoconstriction is specific to renal vessels (Wilcox 1983). The administration of high-chloride solutions and their likely adverse effects on renal function have been studied both in animals (Peng et al. 2012) and human volunteers (Murugan et al. 2010), with the investigators demonstrating a correlation between higher concentrations of pro-inflammatory markers and the development of acute kidney injury. Compared with Ringer lactate—a balanced crystalloid, the administration of NS prolonged the time to the first miction in healthy volunteers (Williams et al. 1999).

It has been suggested by several authors that hyperchloremia developing during NS administration is not exclusively iatrogenic but part of the complex pathophysiology of the given condition (e.g., sepsis). In their experimental animal model of septic shock (induced by infusion of *Escherichia coli* endotoxin), Kellum et al. administered NS for fluid resuscitation. Using this model, the authors demonstrated that exogenous Cl^−^ supplementation may represent but a third of the total increase in serum Cl^−^ concentrations. The remaining two-thirds were ascribed to the activation of mechanisms leading to the different movement of Na^+^ and Cl^−^ ions from the intracellular to extracellular space and vice versa, endothelial injury and albumin extravasation (Williams et al. 1999).

Besides, hyperchloremia may have an anti-inflammatory effect (Kaplan and Kellum 2010) (although if it is together with hypernatremia usually found in metabolic syndrome in people with high dietary intake of sodium chloride it has a rather pro-inflammatory effect (Hucke et al. 2016)), increase intra-abdominal pressure (Gheorghe et al. 2010), and induce a coagulation disorder (Handy and Soni 2008).

### Pathophysiology of hypochloremia

Hypochloremia develops as a result of increased loss of Cl^−^ from the body (gastrointestinal tract (GIT), kidneys) or due to its relative lack during the expansion of the extracellular space by a fluid with low or zero Cl^−^ content (relative hypochloremia). Increased Cl^−^ losses from the GIT are typically associated with massive vomiting. The same losses may occur during extensive gastric content diversion with a nasogastric probe, high output from a small intestine stoma, or, possibly, secretion into the GIT by some types of villous intestinal adenomas. The congenital form of high Cl^−^ losses from the GIT, referred to as congenital chloridorrhea, is a rare occurrence. Primary renal Cl^−^ losses are due to kidney failure or the use of some diuretics (loop diuretics, thiazides, acetazolamide). Some syndromes (e.g., Bartter’s syndrome) are associated with increased urinary chloride excretion (Berend et al. 2012) (Table 2).

**Table 2:** A list of causes of hypochloremia

Mechanism	Site of action	Example
Loss of chlorides	Gastrointestinal tract	Vomiting Gastric drainage Ileostomy with high waste products elimination rates
	Kidney	Diuretics Bartter’s syndrome Gitelman’s syndrome
Excess intake of water (relative to chlorides)	Congestive heart failure	Hypotonic solution infusion
	SIADH	
Excess sodium intake (relative to chlorides)		NaHCO_3_ infusion

*SIADH* the syndrome of inappropriate antidiuretic hormone secretion, *NaHCO*_3_ sodium bicarbonate. Adapted from Bandak G, Kashani KB. Chloride in intensive care units: a key electrolyte. *F1000Res*. 2017; 6:1930

As shown above, Cl^−^ concentrations in typical situations correlate inversely with bicarbonate concentrations; hence a decrease in Cl^−^ (e.g., due to massive vomiting) is associated with increased bicarbonate reabsorption rates resulting in metabolic alkalosis (the decrease in Cl^−^ in glomerular ultrafiltrate reduces the exchange of bicarbonate in the proximal tubule (Na^+^ Cl^−^/HCO_3_^−^ exchanger)). A similar scenario occurs when administering the loop diuretic furosemide with an alkalizing action, which again leads to the development of metabolic alkalosis (Cioccari and Bellomo 2017), in which case the Cl^−^–HCO_3_^−^ antiport is blocked. The situation is quite the opposite in chronic respiratory failure, where the increased Cl^−^ losses through the kidney are secondary, compensatory, with bicarbonate increasingly reabsorbed. The mechanism of hypochloremia development is identical to that of hyperaldosteronism.

In the two cases above, the implications of Cl^−^ concentrations on acid-base balance are intuitive; Cl^−^ losses lead to alkalosis and vice versa. However, in hypochloremia as a consequence of extracellular space expansion with a low Cl^−^ content, the pathophysiological effects may be different requiring a more comprehensive assessment of the acid-base balance disorder. In a theoretical model considering the possibility of mixing the volume of plasma with the same volume of solute-free water (or, possibly, a glucose solution) (Berend et al. 2012), the baseline values of natremia of 140 mmol/L and chloremia of 100 mmol/L will result in concentrations of Na^+^ 70 mmol/L and Cl^−^ 50 mmol/L in the internal milieu (a decrease by 50% in both). However—if simplifying the Stewart approach of acid-base balance in this case (which is admissible)—the SID has changed from 40 to 20 with the difference made up for by bicarbonate. In this scenario, bicarbonate concentrations decrease, followed by what is referred to as dilution acidosis (although, by absolute numbers, hypochloremia is present) (Yunos et al. 2010). An opposite case with similar pathophysiology is the loss of solute-free water from the body, a phenomenon with an alkalizing effect.

Regarding the role of hypochloremia in the pathophysiology of specific clinical entities, it is largely involved in heart failure. Low Cl^−^ concentrations are characteristic of advanced heart failure; some authors have suggested hypochloremia is associated with a reduced left ventricular ejection fraction and elevated levels of cardiac markers. Thus Cl^−^ can serve as an independent predictor of mortality in this patient population (Testani et al. 2016).

In clinical terms, hypochloremia manifests itself by non-specific symptoms noticeable primarily in metabolic alkalosis (if developing) including apathy, confusion, cardiac arrhythmias, and increased muscle irritability (caused by decreased ionized calcium concentrations) (Marchenko et al. 2020).

### The importance of chloride in the composition of crystalloid solutions

Crystalloid solutions are mixtures of electrolytes, water (*aqua pro injectione*), anion buffers, and glucose, which have been used in medicine for almost 190 years. Although their safety has been highlighted as early as 100 years ago (Evevans 1911), it was not until the end of the past millennium that the first studies addressing their safety in clinical practice were published.

Chloride is the main anion in the composition of crystalloids. The first solution employed in the cholera pandemics contained, inter alia, 0.6% NaCl, i.e., a concentration similar to that in the plasma (Awad et al. 2008). The composition of the currently most widely used saline (0.9% NaCl) was published by Hamburger in the late nineteenth century after in vitro experiments with hemolysis. The NaCl concentration of 0.9% was mistakenly considered isotonic (the actual osmolality was 308 mosmol/L, not the reported 285 mosmol/L); nonetheless, the term physiological solution (*normal saline*, NS) quickly gained acceptance as did its clinical use (Reddy et al. 2016). However, NS is non-physiological, for 3 reasons: (i) the concentration of Cl^−^ in the solution is significantly higher than that in the plasma (154 mmol/L); (ii) the solution does not contain other electrolytes present in the plasma, and (iii) it does not contain a buffer to keep the pH within the physiological range. As a result, the SID of NS is zero; hence, the administration of a large volume of NS entails a decrease in plasma SID and, eventually, the development of hyperchloremic metabolic acidosis (Morgan et al. 2004). Balanced solutions became available in the 1930s. Their advantages include lower Cl^-^ concentrations and, consequently, a smaller impact on the acid-base balance. The decrease in chloride content was achieved through its substitution by a metabolizable anion with buffer-like characteristics maintaining a stable pH, i.e., lactate, acetate, maleate, and gluconate (Zadák et al. 2010). Still, chloride content in individual balanced solutions is not uniform (Table 3), with the highest and lower chloride contents being in Ringerfundin and Plasmalyte, respectively. The smaller the chloride content, the higher the content of metabolizable anions. Cl^−^ content may be a consideration when selecting a solution in specific clinical situations based on the SID value (e.g., hypochloremic metabolic alkalosis in protracted vomiting with a high SID).

**Table 3:** Classification of crystalloid solutions (glucose solutions and diluted NS with glucose are not listed)

		Unbalanced solutions		Balanced solutions				
Parameter	Plasma	NS	Ringer	H	RF	PL	Isolyte	Benelyte
Na^+^ [mmol/l]	140	154	147	130	140	140	137	140
K^+^ [mmol/l]	4		4	5	4	5	4	4
Cl^−^ [mmol/l]	104	154	156	125	127	98	110	118
Ca^2+^ [mmol/l]	2.2		2.025	1	2.5			1
Mg^2+^ [mmol/l]	1		1	1	1	1.5	1.5	1
Bicarbonate [mmol/l]	24							
Lactate [mmol/l]	1			27				
Acetate [mmol/l]					24	27	34	30
Malate [mmol/l]					5			
Gluconate [mmol/l]						23		
Glucose [mmol/l]	4.5							55.5
Osmotic concentration [mosmol/l]	285	308	309	276	304	296	286	351
BE	0	− 24	− 24	3	5	26	8	–
pH	7.4	5.3	6.0	6.0	5.5	7.4	7.4	5.5
Na:Cl	1.33:1	1:1	1.06:1	1.18:1	1.1:1	1.43:1	1.24:1	1.19:1

*NS* 0.9% (NaCl) normal saline, *H* Hartman’s solution, *RF* Ringerfundin, *PL* Plasmalyte, *Na* sodium, *K* potassium, *Cl* chloride, *Ca* calcium, *Mg* magnesium, *BE* base excess, *mmol*/*L* millimole per liter, *mosmol*/*L* milliosmole per liter. Values given for plasma are means

### Clinical relevance of fluid therapy-induced iatrogenic hyperchloremia

The choice of a crystalloid solution should be based on knowledge of the pathophysiology of the disease and the current state of the patient’s internal milieu; the rationale for fluid therapy should also be taken into account.

Scheduled surgical procedures are undertaken mostly in patients with normal renal function and an internal milieu within the physiological range; hence, increased attention should be given to potential changes induced by crystalloid solutions. In their study with 22,851 patients who had noncardiac surgery with normal pre-operative laboratory values and normal renal function, McCluskey et al. documented acute postoperative hyperchloremia (Cl^−^ > 110 mmol/L) in 22% of those enrolled. These patients had a higher incidence of renal failure, and hyperchloremia was an independent predictor of 30-day mortality (risk ratio 2.05; 95% confidence interval 1.62–2.59) (McCluskey et al. 2013). A similar conclusion was drawn by a study enrolling cardiac surgical patients (Van Regenmortel et al. 2016). Likewise, utmost attention is to be paid to renal transplant recipients (Romagnoli and Ricci 2019). Although patients receiving NS have been shown to have a higher incidence of hyperchloremia, acidosis, and hyperkalemia (Weinberg et al. 2017), these changes have been shown to have a clinically significant impact on the deterioration of graft function (Wan et al. 2016). By implication, NS administration leads to intraoperative iatrogenic changes in plasma composition. To what extent this phenomenon affects the long-term outcome of the patient is not clear yet. However, attention should also be given to internal milieu fluctuations (just as with blood pressure or blood oxygen saturation) even more so as they may have occurred due to the unselective administration of fluids—substances which are one of the most widely used in our practice. Each episode of hyperchloremia harms the body (just as hypotension and hypoxia).

In a study with 15,802 intensive care patients, Self et al. found lower rates of renal failure, use of dialysis, and lower total mortality in those receiving fluid therapy with balanced crystalloid solutions (Self et al. 2018). Regarding the development of hyperchloremia, solutions associated with a certain risk are not only those used for volume replacement as part of initial circulatory resuscitation (Bandak and Kashani 2017; Sen et al. 2017) but, also, solutions employed for the dilution and reconstitution of the drugs administered (e.g., antibiotics), vitamin therapy, flushing catheters and sets, and so on (Van Regenmortel et al. 2019; Van Regenmortel et al. 2018). The effect of the implementation of new strategies reducing the rates of administering high-chloride solutions was well documented in the study of Yuons et al., where the implementation of control measures in fluid therapy (administration of Hartman’s solution, Plasmalyte, and 20% albumin with low Cl^−^ content instead of the uncontrolled administration of various types of solutions) was followed by a statistically significant decrease in the rates of acute renal failure and the use of dialysis (Yunos et al. 2012).

## Conclusion

Chloremia and its disturbances should be evaluated in the whole clinical context. From the physiological point of view, chloride ions represent one of the fundamental players in acid-base balance and have a profound effect on renal physiology. On the other hand, the chloride load in fluid therapy is not the sole mechanism that increases chloremia. The underlying disease usually contributes significantly. Perioperative fluid therapy should be always based on physiological concepts and reflecting patient-specific underlying pathophysiology and clinical circumstances.

## Data Availability

Not applicable.

## Abbreviations

CO_2_: Carbon dioxide
H_2_CO_3_: Carbonic acid
HCO_3_^−^: Bicarbonate anion
H^+^: Hydrogen cation
Hb^−^: Hemoglobin
HHb: Hydrogen cation bound to hemoglobin
Cl^-^: Chloride anion
SIADH: Syndrome of inappropriate antidiuretic hormone secretion
NaHCO_3_: Sodium bicarbonate
NS: Normal saline
H: Hartman’s solution
RF: Ringerfundin
PL: Plasmalyte
Na^+^: Sodium cation
K^+^: Potassium cation
Ca^2+^: Calcium cation
Mg^2+^: Magnesium cation
BE: Base excess
Cl^-^_[in]_: Intracellular concentration of chloride
SID: Strong ion difference
mmol/L: Millimole per liter
mosmol/L: Milliosmole per liter
mV: Millivolt
GIT: Gastrointestinal tract
